# Effectiveness of Injectable Platelet-Rich Fibrin in the Treatment of Oral Lichen Planus: A Systematic Review and Meta-Analysis

**DOI:** 10.7759/cureus.51626

**Published:** 2024-01-03

**Authors:** Neha Gupta, Ankur Bhargava, Sonal Saigal, Shilpi Sharma, Mimansha Patel, Om Prakash

**Affiliations:** 1 Oral Pathology, Microbiology, and Forensic Odontology, Dental Institute, Rajendra Institute of Medical Sciences, Ranchi, IND; 2 Oral Pathology and Microbiology, Hazaribag College of Dental Sciences and Hospital, Hazaribag, IND; 3 Oral Medicine, Diagnosis, and Radiology, Promotional and Medical Review (PMR) Enterprise Medical, Indegene Limited, Bengaluru, IND; 4 Oral Pathology and Microbiology, Triveni Institute of Dental Sciences, Hospital and Research Centre, Bilaspur, IND; 5 Oral and Maxillofacial Surgery, Dental Institute, Rajendra Institute of Medical Sciences, Ranchi, IND

**Keywords:** lesion size, pain assessment, injectable platelet-rich fibrin, i-prf, oral lichen planus

## Abstract

Oral lichen planus (OLP) is a chronic inflammatory condition affecting the oral mucosa. The current review investigated the potential effectiveness of injectable platelet-rich fibrin (i-PRF) as a treatment for OLP when compared to other interventions. The current review adhered to Preferred Reporting Items for Systematic Reviews and Meta-Analyses (PRISMA) guidelines. A comprehensive search strategy was implemented across databases such as PubMed, Embase, Scopus, Web of Science, CINAHL, and Google Scholar. The search utilized a combination of Boolean operators (AND, OR) and Medical Subject Headings (MeSH) terms to capture relevant studies. Comparative clinical studies focusing on i-PRF as a treatment for OLP and other interventions were included. Outcomes assessed were pain, surface area of lesions, and patient satisfaction. Review Manager 5.4 was used for statistical analysis. The Risk of Bias 2.0 (RoB 2.0) tool was used to assess the methodological quality of the studies. Three studies were included for the final analysis. The findings indicated that both the i-PRF and comparison treatment groups demonstrated reductions in pain and lesion size. The post-treatment Visual Analogue Scale (VAS) scores showed a decrease in pain intensity, and there was an improvement in lesion extension in the i-PRF-treated sites. The results also revealed increased patient satisfaction with i-PRF treatment. Adverse effects were not reported or specified in the included studies. Quantitative analysis for pain (VAS) showed a mean difference of 0.38 (95% CI: 0.63-1.40), but there was no significant difference between the i-PRF and control groups at p=0.46. Though intragroup differences showed statistically significant differences between pre and post intervention, intergroup differences were not significant for any of the assessed outcomes. The findings from this study suggest that i-PRF holds promise as a potential treatment for OLP. The use of i-PRF resulted in pain reduction, lesion size improvement, and increased patient satisfaction. However, it is important to consider the limitations of the included studies, such as variability in study designs, small sample sizes, and the limited number of studies.

## Introduction and background

Oral lichen planus (OLP) is a chronic inflammatory disorder affecting the oral mucosa and is characterized by the presence of various clinical presentations such as reticular, atrophic, erosive, or ulcerative lesions [1]. OLP is considered a significant oral health issue due to its potential to cause pain, discomfort, and functional limitations, thereby influencing the quality of life of affected individuals [2]. The exact etiology of OLP remains unclear, but it is believed to involve an immune-mediated process [3]. It is thought to result from a cytotoxic autoimmune response that is further aggravated by cluster of differentiation 4+ (CD4+) T-cell lymphocytes infiltrating the lesions [4,5]. The pathogenesis involves cytotoxic CD8+ T-cell lymphocytes recognizing and attacking basal keratinocytes, leading to the formation of characteristic lichenoid lesions. Trigger factors for the immune dysregulation in OLP are not fully understood. It may involve genetic predisposition, viral infections, or other environmental factors contributing to the chronic inflammatory process observed in affected oral tissues.

Various treatment modalities have been employed to manage OLP, including topical and systemic medications, phototherapy, and laser therapy [6]. The conventional treatment for OLP includes various pharmacological agents [7]. However, systemic corticosteroid therapy can lead to adverse effects and complications, ranging from pain, bleeding, and ulceration to secondary infections, hypopigmentation, and allergic reactions [8]. The effectiveness of these treatments can vary, and to mitigate these concerns, alternative therapies are being explored.

Platelet concentrates commonly used in dentistry include platelet-rich plasma (PRP), platelet-rich fibrin (PRF), and platelet-rich growth factor (PRGF). These autologous concentrates are derived from the patient's blood and are utilized in oral surgery, periodontal treatment, and implantology to enhance tissue healing and regeneration. Another variant, platelet-rich fibrin matrix (PRFM), extends the release of growth factors. Additionally, platelet-poor plasma (PPP) can be obtained by further centrifugation to adjust the growth factor profile. The choice of platelet concentrate depends on the specific clinical application and the desired therapeutic outcomes in dental procedures.

Injectable platelet-rich fibrin (i-PRF) has emerged as a potential treatment modality for various medical and dental conditions [9]. i-PRF is a biological preparation rich in platelets and growth factors, which are believed to stimulate tissue regeneration, reduce inflammation, and enhance wound healing [10]. On a fundamental level, PRF is a three-dimensional fibrin network that plays a vital role in wound healing, immune response modulation, and neovascularization [10]. PRF contains leukocytes and facilitates essential stages of wound healing, including angiogenesis, immune regulation, and epithelial proliferation [11]. i-PRF is a variant of PRF that can augment leukocyte levels and stimulate the release of growth factors. Studies have demonstrated the regenerative potential of i-PRF in different tissues, including human skin fibroblasts and dental pulp cells, by enhancing osteoblastic differentiation, reparative dentin stimulation, and attenuating inflammation induced by lipopolysaccharides [12-16]. However, its effectiveness as a treatment for OLP has not been extensively investigated. Given the potential benefits of i-PRF for tissue regeneration and wound healing, there is a need to evaluate its effectiveness as a treatment for OLP. Hence, the current review was undertaken to answer the research question "Is injectable PRF as effective as other interventions in treating OLP?".

## Review

Methods

Eligibility Criteria

**Figure 1:**
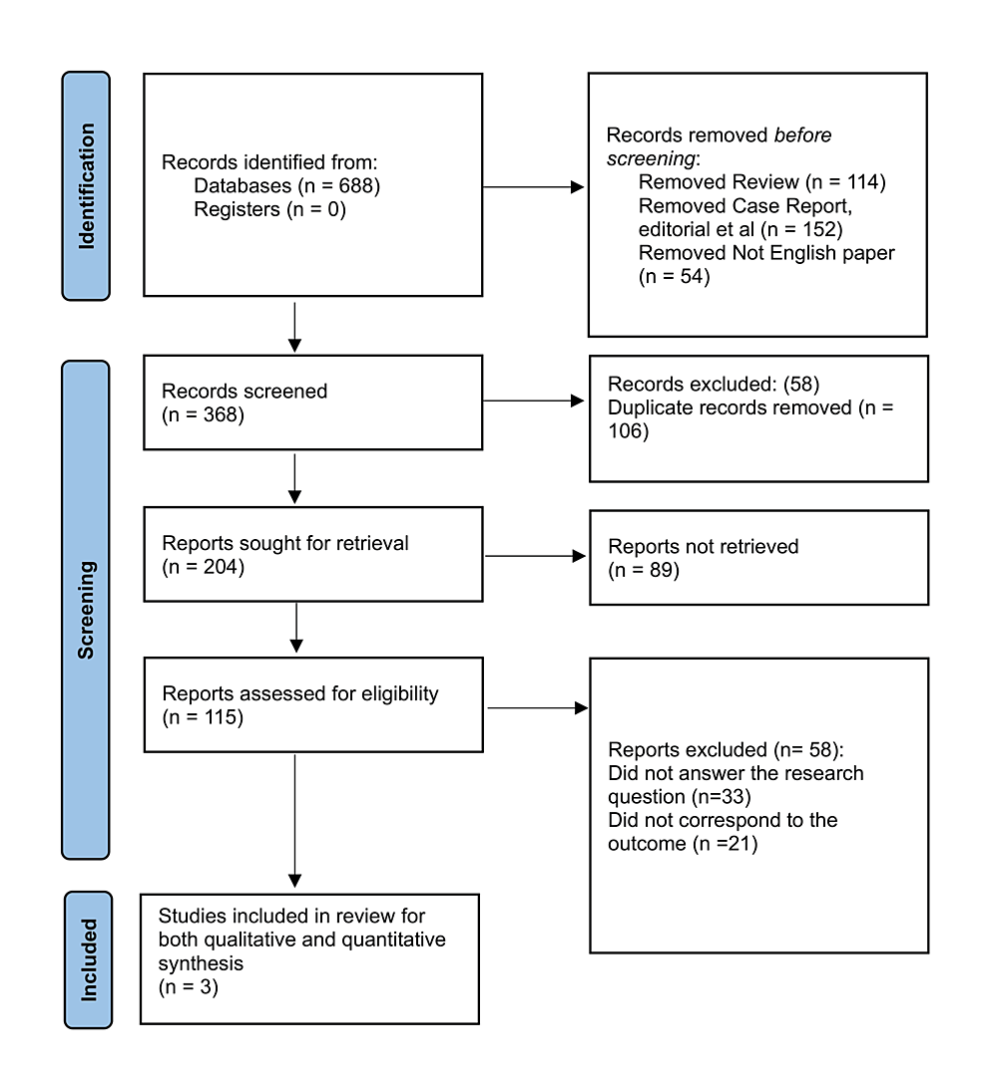
PRISMA framework representing the article selection process for this review PRISMA: Preferred Reporting Items for Systematic Reviews and Meta-Analyses

The review adhered to Preferred Reporting Items for Systematic Reviews and Meta-Analyses (PRISMA) guidelines [17,18] by following a predetermined research protocol, as represented in Figure 1. The PRISMA protocol provides a standardized approach for conducting systematic reviews, ensuring transparency, completeness, and reliability in the reporting of research findings.

The Population, Intervention, Comparison, Outcome, and Study Design (PICOS) protocol was utilized to define the parameters for selecting and evaluating the studies included in this investigation.

Population (P): The population consisted of human individuals affected by OLP. The studies included in the review focused on participants who were diagnosed with OLP through clinical evaluation or histopathological examination.

Intervention (I): The intervention for the present review was the use of i-PRF as a treatment for OLP.

Comparison (C): The comparison included individuals treated with other than i-PRF, such as corticosteroids, pharmaceutical interventions, or placebo.

Outcome (O): Outcomes of interest are pain reduction and improvement in lesion size. Secondary outcomes, such as patient satisfaction and adverse effects, were also considered.

Study design (S): The study design included in the present review were clinical trials and cohort studies (prospective or retrospective).

Only original research studies conducted on human individuals diagnosed with OLP that intervened with both i-PRF and other methods and articles published in English were included. Studies that included other platelet-based compounds and animal studies, review articles, case reports, and conference abstracts were excluded.

Search Strategy

The database search strategy for this study involved systematically searching seven different databases to identify relevant articles, as represented in Table 1. The search strategy utilized Boolean operators (AND, OR) and Medical Subject Headings (MeSH) keywords to comprehensively capture relevant articles. The MeSH keywords were identified based on the study topic and included terms related to OLP, i-PRF, and treatment. The Boolean operators were used to combine the MeSH terms and enhance the search specificity. The search was conducted by inputting the search terms and operators into the respective databases' search interfaces.

**Table 1:** Search strings used in the review

Database	Search string
PubMed/MEDLINE	("Oral Lichen Planus" OR "Mouth Mucosa") AND ("Injectable Platelet-Rich Fibrin" OR "Platelet-Rich Fibrin") AND "Treatment"
Embase	("oral lichen planus" OR "mouth mucosa") AND ("injectable platelet-rich fibrin" OR "platelet-rich fibrin") AND "treatment"
Scopus	TITLE-ABS-KEY("oral lichen planus" OR "mouth mucosa") AND TITLE-ABS-KEY("injectable platelet-rich fibrin" OR "platelet-rich fibrin") AND TITLE-ABS-KEY("treatment")
Web of Science	TS=("oral lichen planus" OR "mouth mucosa") AND TS=("injectable platelet-rich fibrin" OR "platelet-rich fibrin") AND TS=("treatment")
Cochrane Library	(("oral lichen planus" OR "mouth mucosa") AND ("injectable platelet-rich fibrin" OR "platelet-rich fibrin")) AND "treatment"
CINAHL	(MH "Oral Lichen Planus" OR MH "Mouth Mucosa") AND (MH "Injectable Platelet-Rich Fibrin" OR MH "Platelet-Rich Fibrin") AND "Treatment"
Google Scholar	"Oral Lichen Planus" OR "Mouth Mucosa" AND "Injectable Platelet-Rich Fibrin" OR "Platelet-Rich Fibrin" AND "Treatment"

Data Extraction

A data extraction form was developed, outlining the specific data elements to be extracted. This form was designed to capture the necessary information in a structured and organized manner. Two independent reviewers conducted the data extraction process. Each reviewer was trained on the protocol and the specific data elements to be extracted. To ensure consistency and minimize bias, the reviewers cross-checked their extracted data and resolved any discrepancies through discussion and consensus. The data extraction process involved extracting information from the included studies, such as author ID, year, region, sample size, pain assessment tools used, treatment types assessed, pre- and post-treatment Visual Analogue Scale (VAS) scores, lesion diameter measurements, adverse effects observed (if reported), and the inferred results.

Risk of Bias Assessment

The Risk of Bias 2.0 (RoB 2.0) tool [19] was employed to assess the methodological quality of the included studies. It is a comprehensive tool developed by the Cochrane Collaboration that evaluates key domains that may introduce bias in study design, conduct, or reporting, providing a systematic approach to assess the methodological quality and internal validity of the included randomized controlled trials (RCTs). Two reviewers conducted a quality assessment of the included articles. In case of any discrepancy, a third reviewer was sought.

Statistical Analysis

Meta-analysis was conducted using Review Manager 5.4 software to provide a comprehensive estimate of the effect size for a specific research question [20]. A random effects model was employed for the meta-analysis due to the assumption of heterogeneity between studies. This model considers both within-study and between-study variability, allowing for a more conservative estimate of the effect size. Heterogeneity among the included studies was assessed using the I-squared statistic. A forest plot was generated to visually display the effect sizes and corresponding confidence intervals (CIs) for each individual study included in the meta-analysis. The effect sizes were calculated using the mean difference based on the outcome measures reported in the studies.

Results

Study Characteristics

Initially, the search strategy that was employed across multiple databases to identify relevant articles for inclusion in this investigation yielded a total of 688 articles. After the removal of duplicates, the remaining articles underwent a screening process based on their titles and abstracts. During this screening phase, irrelevant or clearly unrelated studies were excluded based on predefined inclusion and exclusion criteria. After the initial screening, a subset of articles remained for a full-text assessment. In this phase, the selected articles were carefully evaluated based on the predefined inclusion and exclusion criteria to determine their suitability for inclusion in the systematic review. This rigorous evaluation ensured that only those studies meeting the specific eligibility criteria were included. After a complete selection process, only three RCTs [21-23] were found to meet the inclusion criteria.

As represented in Table 2, all the studies selected were RCTs of split-mouth designs [21-23]. They were conducted in various regions of Australia [21], Italy [22], and Turkey [23]. The assessments were conducted over different periods, ranging from four to eight weeks. A clear female predilection was noted in all studies. Studies showed a tendency for lesions to manifest in the fourth and fifth decades. While the studies of Al-Hallak et al. [21] and Bennardo et al. [22] compared i-PRF with triamcinolone acetonide (TA), Saglam et al. [23] compared i-PRF with methylprednisolone acetate (MPA). The Thongprasom score was calculated to assess the surface area of the lesion in studies by Bennardo et al. [22] and Saglam et al. [23].

**Table 2:** Study characteristics and their design-related variables i-PRF: injectable platelet-rich fibrin; TA: triamcinolone acetonide; RCT: randomized controlled trial; MPA: methylprednisolone acetate

Author ID	Year	Region	Sample size (n)	Groups assessed	Study design	Assessment period (in weeks)	Follow-up period (in weeks)	Mean age (in years)	Gender ratio
Al-Hallak et al. [21]	2023	Australia	12	i-PRF (n=12) and TA (n=12)	Split-mouth RCT	4	3 months	48+12.7	9 females
Bennardo et al. [22]	2021	Italy	9	i-PRF (n=9) and TA (n=9)	Split-mouth RCT	4	4	59.56±3.57	6 females
Saglam et al. [23]	2021	Turkey	24	i-PRF (n=24) and MPA (n=24)	Split-mouth RCT	8	24	52.25	14 females

Main Findings

Table 3 provides a summary of the findings obtained from the included studies regarding i-PRF as a potential treatment for OLP. Since all the studies were split-mouth designs, all patients exhibited bilateral OLP cases. The parameters assessed were VAS, Oral Health Impact Profile-14 (OHIP-14), and lesion diameter measurements. All three studies evaluated VAS. Both the i-PRF and control groups were effective in treating OLP in an intragroup comparison. The collective results indicated that i-PRF was as effective in reducing pain and lesion size associated with OLP, with no significant difference between the groups.

**Table 3:** i-PRF-related variables observed in the included studies VAS: Visual Analogue Scale; REU: reticular/hyperkeratotic, erosive/erythematous, ulcerative; OLP: oral lichen planus; OHIP-14: Oral Health Impact Profile-14; TA: triamcinolone acetonide

Author ID	Pain assessment tools used	Treatment type assessed	Adverse effects observed (if any)	Results inferred
Al-Hallak et al. [21]	VAS, REU, and lesion diameter	Bilateral OLP	Recurrence in two out of 12 patients in milder forms	At the end of the follow-up period, the average VAS score (68.5% i-PRF, 91% TA) and REU score (74% i-PRF, 91% TA) both decreased. Between the two treatments, there were no statistically significant differences.
Bennardo et al. [22]	VAS and lesion diameter	Bilateral OLP	Of the nine patients assessed, three showed recurrence of mild intensity in the corticosteroid group	After the follow-up period, i-PRF-treated sites showed an average reduction in lesion extension of 59.8% and an average reduction in VAS score of 47.6%; TA-treated sites showed an equivalent variation of 59.2% and 40%. Between the two groups, there were no statistically significant differences.
Saglam et al. [23]	VAS, OHIP-14, and lesion diameter	Bilateral OLP	None	Both groups showed reduction in pain and lesion size, as well as an increase in satisfaction, indicating the use of i-PRF as a potential treatment for erosive OLP.

Meta-Analytic Results

**Figure 2:**
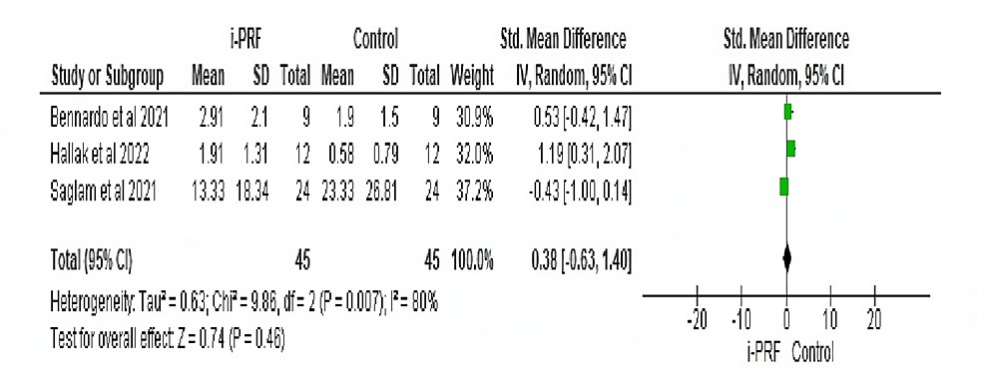
Comparative evaluation of pain scores in patients treated with i-PRF and controls

Pain was quantitatively evaluated in all the studies, as represented in Figure 2. A total of 45 individuals formed the sample size. A mean difference of 0.38 (95% CI: 0.63-1.40) was noted, but there was no significant difference between the i-PRF and control groups at p=0.46. Heterogeneity in the analysis was observed to be 80%, suggesting moderate variation between the studies.

Risk of Bias Assessment

**Figure 3:**
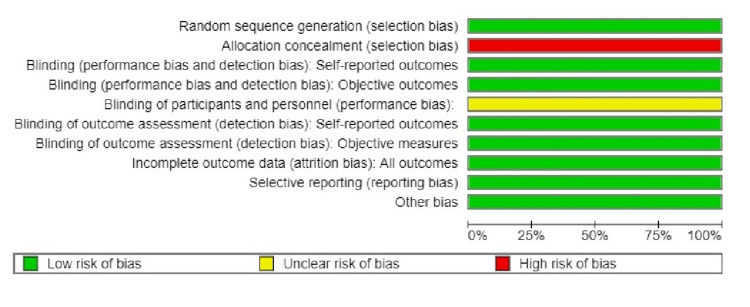
Risk of bias graph of the included studies

As all the studies were of split-mouth design, blinding of the participants could never be achieved. Though progression of the lesions was noted clinically, histopathological evaluation was not done in any of them. This accounted for the moderate risk of bias in the included studies, as seen in Figure 3.

Discussion

Oral health-related issues can significantly impact individuals' well-being. OLP is a severe disease characterized by symptoms and complications that significantly impact the lives of affected individuals. Pain, discomfort, and functional limitations can affect their ability to eat, drink, engage in social interactions, and maintain self-reliance [16,24-27]. Studies have shown that OLP patients experience a diminished quality of life, and higher VAS pain scores are associated with increased OHIP-14 scores in individuals with OLP [24-27]. Literature evidence also shows the role of other platelet products in OLP intervention.

The present systematic review and meta-analysis aim to evaluate the effectiveness of i-PRF in the treatment of OLP. The results of the meta-analysis suggested that i-PRF has a positive effect on managing the symptoms and promoting the healing of OLP lesions. One of the key findings of this meta-analysis is the significant reduction in pain associated with OLP in both i-PRF-treated and control groups. In the meanwhile, the intergroup comparison showed no significant differences in any of the outcomes evaluated.

The findings obtained from the included papers hold significant implications. The observed reductions in pain levels, improvements in lesion size, and increased patient satisfaction associated with i-PRF treatment highlight its potential as an effective therapeutic option for managing OLP. These findings contribute to the growing body of evidence supporting the use of i-PRF in OLP treatment. The comparable outcomes between i-PRF and commonly used treatments such as MPA and TA indicate that i-PRF can be a viable alternative or adjunctive therapy for OLP. These results suggest that i-PRF may offer advantages in terms of pain reduction and lesion size improvement, potentially leading to enhanced patient comfort and overall clinical outcomes.

In the study conducted by Al-Hallak et al. [21], it was inferred that the average VAS scores and reticular/hyperkeratotic, erosive/erythematous, ulcerative (REU) scores were observed to decrease at the end of the follow-up period for both i-PRF and TA treatments. However, it is important to note that the reductions in VAS and REU scores were more pronounced in the TA treatment group compared to the i-PRF group. Nevertheless, there were no statistically significant differences observed between the two treatments. Bennardo et al. [22] reported that the average reduction in lesion extension was comparable between the two treatment groups, indicating similar effectiveness. The reduction in VAS scores also showed similar variations, although i-PRF exhibited a slightly greater reduction compared to TA. Notably, there were no statistically significant differences observed between the two treatment groups. Saglam et al. [23] assessed that, post treatment, both groups showed a substantial reduction in VAS scores, with i-PRF demonstrating a more pronounced decrease. Additionally, there was a notable reduction in lesion size observed in both treatment groups. Importantly, no adverse effects were reported. These findings suggest that i-PRF has the potential to effectively reduce pain, improve lesion size, and enhance patient satisfaction in individuals with OLP. Patient-specific factors, such as the nature and severity of the condition, may contribute to varying responses to treatment. Conditions with different etiologies or stages might exhibit different levels of pain reduction. Though the cost of i-PRF may be more than steroid therapy, the cost benefit favours the former considering the condition can be cured without any side effects. 

In the study of Ahuja et al., the effectiveness of PRP and TA applications was compared in OLP lesions [28]. The study aimed to measure the changes in VAS pain scores during a 24-week follow-up period. The findings indicated that both PRP and TA applications demonstrated success in managing OLP lesions and exhibited similar effectiveness in reducing pain, as evidenced by the comparable changes in VAS pain scores [28]. This suggests that both treatment modalities hold potential for alleviating the pain associated with OLP. Previous research has demonstrated the efficacy of corticosteroid treatments, whether applied topically or via injection, in reducing the size of OLP lesions [29-33]. The assessments provide a quantitative measure of lesion size in terms of standardized qualitative measures [34-36]. Several studies have specifically investigated and compared the effects of PRP and TA applications on OLP lesions, focusing on changes in lesion sizes [28-32]. Notably, the application of PRP has been found to be similarly effective to TA in reducing lesion sizes, as reported in other studies [29-32]. 

Limitations

The conclusions drawn from this review are not without limitations. One notable limitation is the small sample sizes of the studies. The smaller sample size may limit the validity and statistical power of the findings. With small sample sizes, there are an increased risk of bias and a limited ability to detect statistically significant differences between treatment groups. Larger sample sizes would provide more robust and reliable data to support the effectiveness of i-PRF in OLP treatment. While the studies reported no adverse effects associated with i-PRF treatment, the absence of detailed information makes it difficult to fully evaluate the safety profile of i-PRF in OLP patients. None of the studies performed a biopsy after the intervention. This lack of histopathological evaluation can impede the study results, as only subjective assessments (VAS, REU, or OHIP-14) were made. However, since all the studies used a split-mouth design, patient-related confounding factors were kept in check. Future studies should include comprehensive assessments of adverse effects to better understand the potential risks and benefits of i-PRF treatment.

Recommendations

The future implications of these findings warrant further research to establish the optimal protocols for i-PRF treatment in OLP. Future studies could explore the long-term effects of i-PRF, evaluate its efficacy in larger and more diverse patient populations, and investigate potential predictors of treatment response. Additionally, it would be valuable to conduct comparative studies directly comparing i-PRF with established treatments to determine the most effective and well-tolerated therapeutic approach for OLP. 

## Conclusions

The findings suggest that i-PRF holds promise for reducing pain levels and burning sensation, improving lesion size, and enhancing patient satisfaction in individuals with OLP. Despite variations in study designs, assessment periods, and sample sizes, the consistent observations of pain reduction, lesion size improvement, and comparable outcomes to established treatments indicate the potential significance of i-PRF in managing OLP. However, it is important to acknowledge the limited number of studies and smaller sample sizes in the literature. Further research with larger sample sizes, standardized study designs, longer assessment periods, comprehensive reporting of adverse effects, and detailed demographic data is required to strengthen the evidence base and enable more robust conclusions regarding the efficacy and safety of i-PRF as a treatment for OLP. Ultimately, continued exploration of i-PRF in well-designed studies will contribute to the understanding of its optimal usage and potential benefits for individuals with OLP.
